# Supplementary material for: Routing algorithms as tools for integrating social distancing with emergency evacuation
Source: Sci Rep. 2021 Oct 4;11:19623. doi: 10.1038/s41598-021-98643-z (PMC8490376; doi:10.1038/s41598-021-98643-z)
Supplement: Supplementary file 1 — Supplementary Information. [file 41598_2021_98643_MOESM1_ESM.pdf]

Supplementary Information

Routing algorithms as tools for integrating social distancing with emergency evacuation

Yi-Lin Tsai[1,*], Chetanya Rastogi[2], Peter K. Kitanidis[1,3,4], and Christopher B. Field[3,5,6]

[1]Department of Civil and Environmental Engineering, Stanford University, Stanford, CA, USA

[2]Department of Computer Science, Stanford University, Stanford, CA, USA

[3]Woods Institute for the Environment, Stanford University, Stanford, CA, USA

[4]Institute for Computational and Mathematical Engineering, Stanford University, Stanford, CA, USA

[5]Department of Biology, Stanford University, Stanford, CA, USA

[6]Department of Earth System Science, Stanford University, Stanford, CA, USA

*Corresponding author: Yi-Lin Tsai (yilin2@stanford.edu)

Supplementary Table S1: Summary of datasets[50,51].

Dataset	Population	# of Houses	Source
1	52	20	First 20 locations in A-n36-k5
2	83	35	All locations in A-n36-k5
3	126	52	A-n53-k7
4	168	68	A-n69-k9

Routing experiments

This section included all of the routing figures generated from our DNN-based and non-DNN solutions. In each figure, a legend box shows the detailed information about that experiment. For example, in Supplementary Fig. S1, the legend box of the DNN-based Solution is in the format of "R0, #14, c 37/64, t: 1.79 hours". R0 is the first route, R1 is the second, etc. "#14" means that an emergency vehicle visits 14 houses in that route. "c 37/64" indicates that the emergency vehicle whose passenger capacity is 64 people picks up 37 people in route R0. Finally, "t: 1.79 hours" means that the emergency vehicle spends 1.79 hours in route R0.

To make the routing pattern easy to read, we hide the first and the last line going from and back to the black square, which represents the transition point for transit to the depot. For each experiment, we showed the total time and the number of routes at the top of each figure. All of the figures in this supplementary information section are for vehicle capacity = 64, 32, 16, 8, 4, or 2 people per emergency vehicle and transit time = 0.

In our study, we used the typical CVRP setting, meaning the emergency vehicle would only visit each house once. For cases where vehicle capacity was 2, to deal with some houses having more than two people, we split the simulations into two parts. In Part I, the vehicle can take up to two people from each household, as shown in Supplementary Fig. S6. People left in each house were picked up in Part II, as shown in Supplementary Fig. S7.

Dataset 1: 52 people and 20 houses

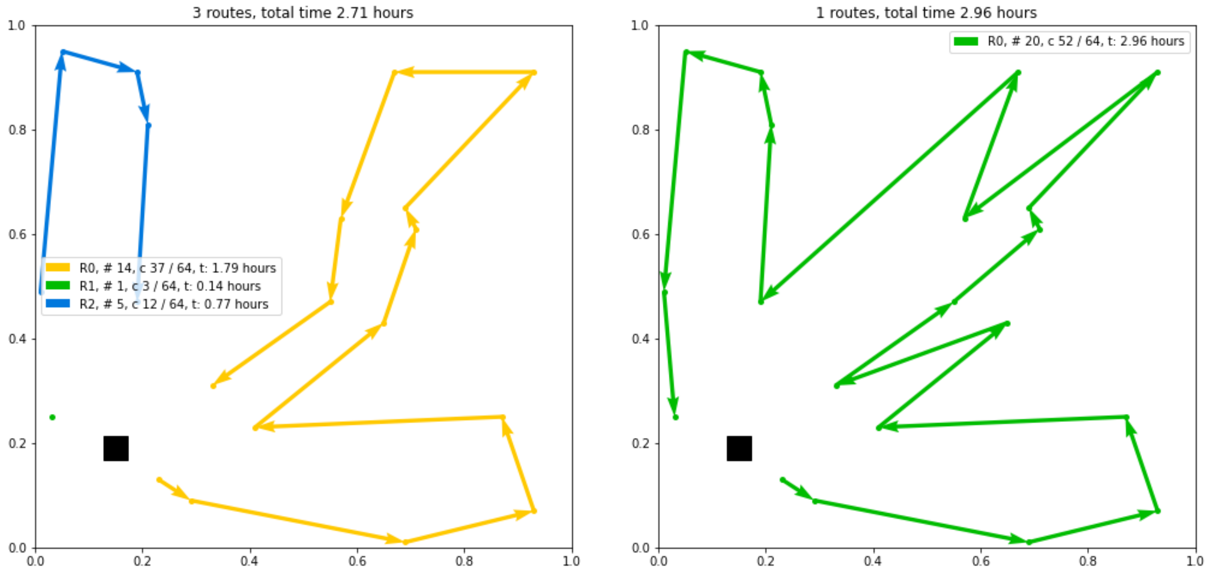

(a) DNN-based Solution

(b) Non-DNN Solution

Supplementary Fig. S1: Social distancing policy: passenger capacity=64 people.

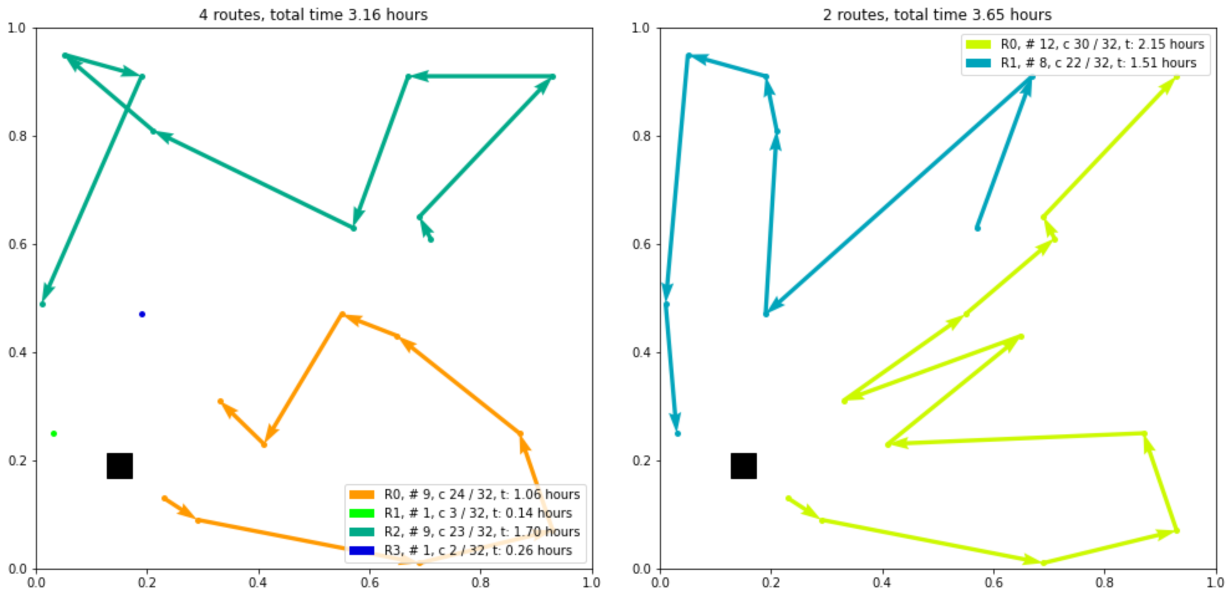

(a) DNN-based Solution

(b) Non-DNN Solution

Supplementary Fig. S2: Social distancing policy: passenger capacity=32 people.

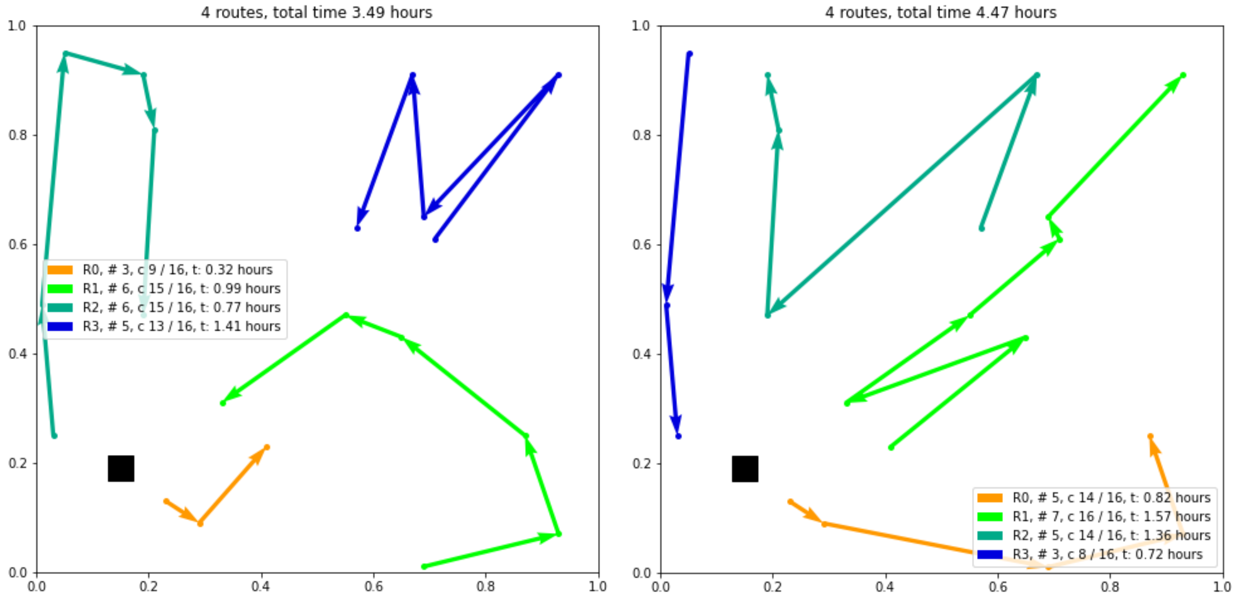

(a) DNN-based Solution

(b) Non-DNN Solution

Supplementary Fig. S3: Social distancing policy: passenger capacity=16 people.

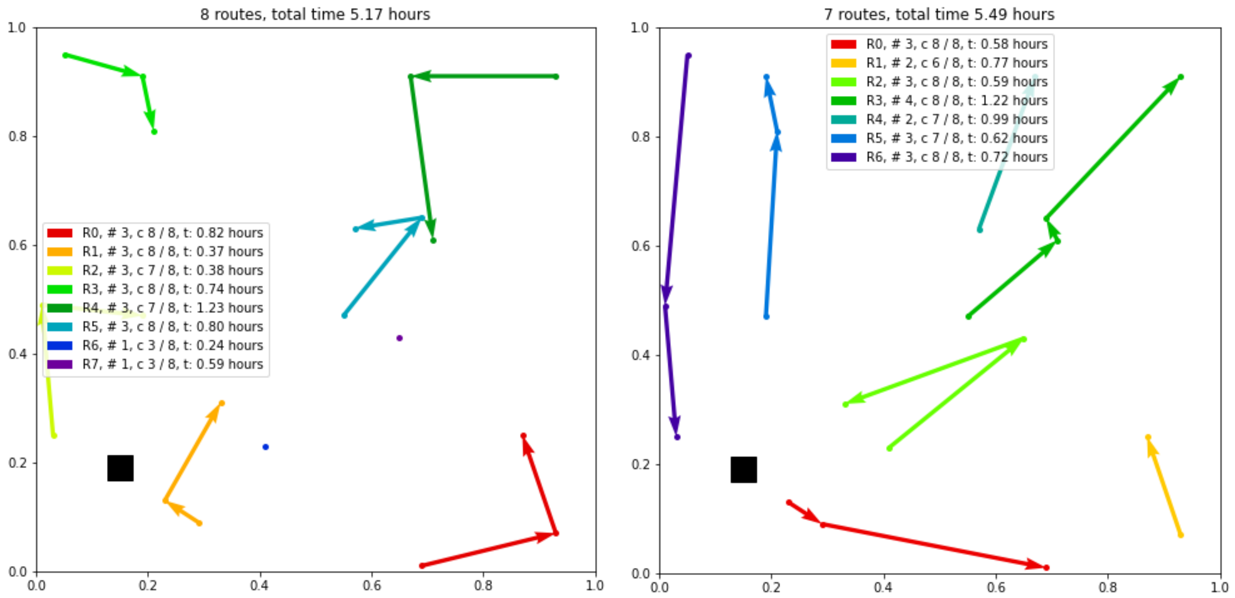

(a) DNN-based Solution

(b) Non-DNN Solution

Supplementary Fig. S4: Social distancing policy: passenger capacity=8 people.

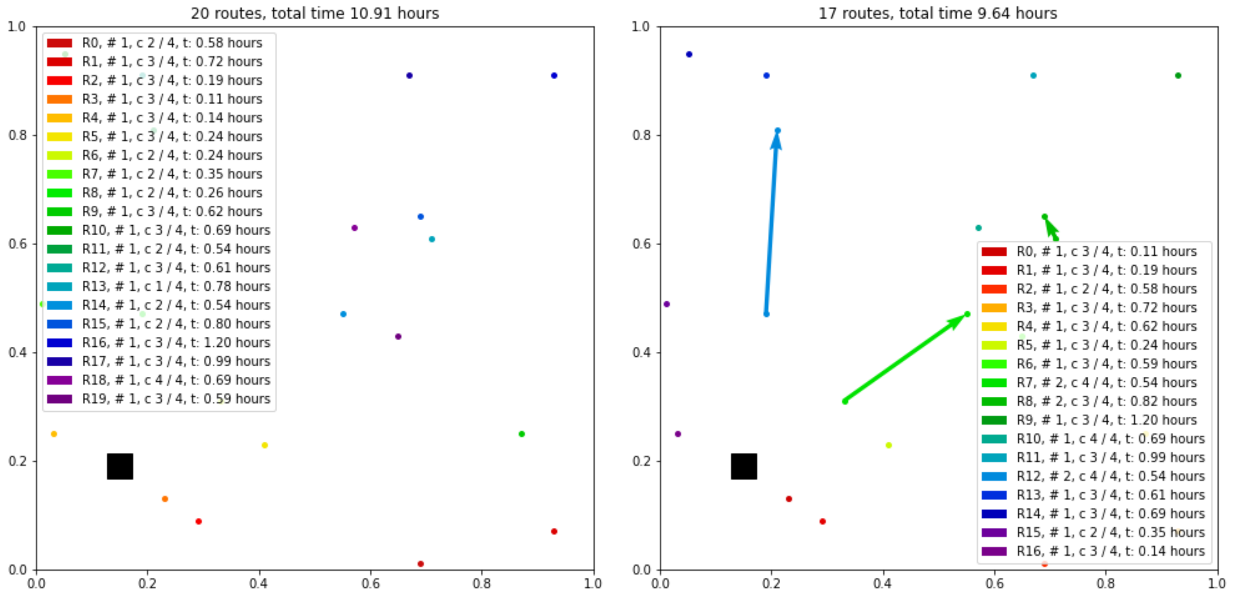

(a) DNN-based Solution

(b) Non-DNN Solution

Supplementary Fig. S5: Social distancing policy: passenger capacity=4 people.

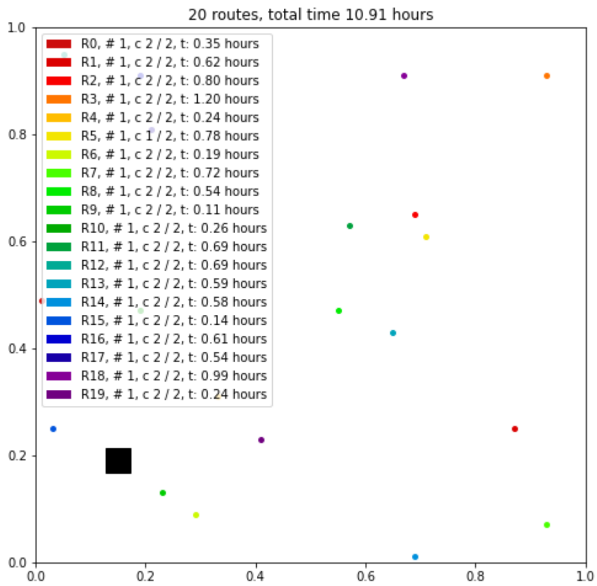

(a) DNN-based Solution

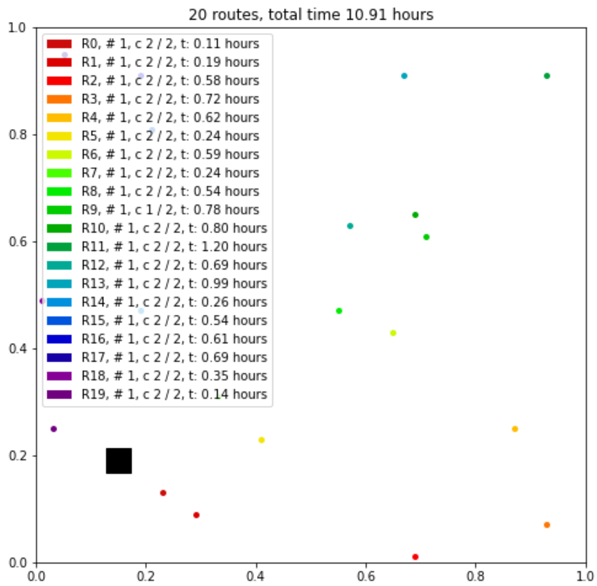

(b) Non-DNN Solution

Supplementary Fig. S6: Social distancing policy: passenger capacity=2 people, Part I.

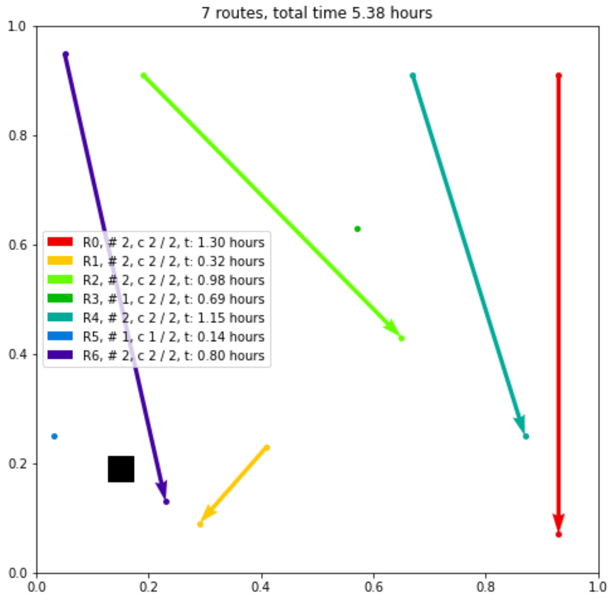

(a) DNN-based Solution

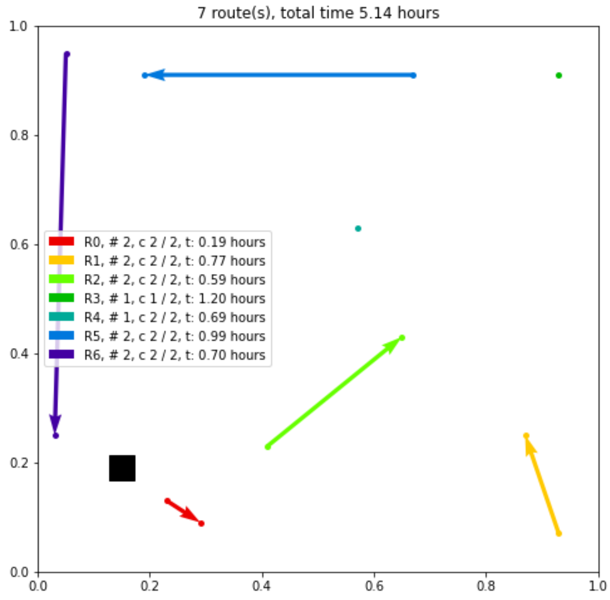

(b) Non-DNN Solution

Supplementary Fig. S7: Social distancing policy: passenger capacity=2 people, Part II.

Dataset 2: 83 people and 35 houses

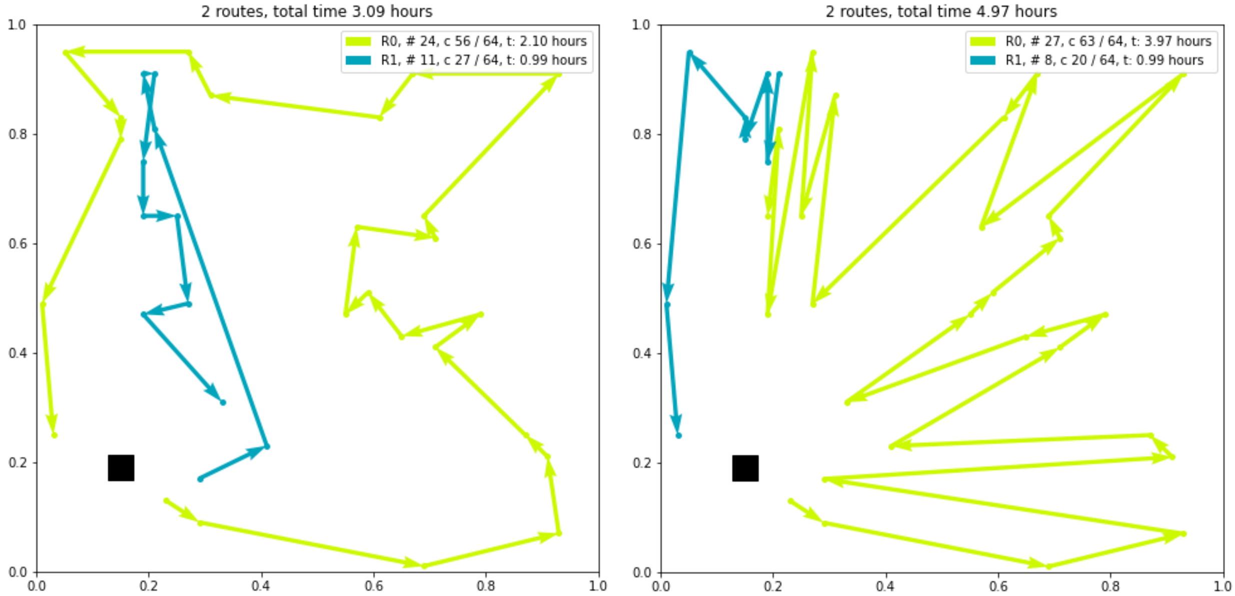

(a) DNN-based Solution (b) Non-DNN Solution

Supplementary Fig. S8: Social distancing policy: passenger capacity=64 people.

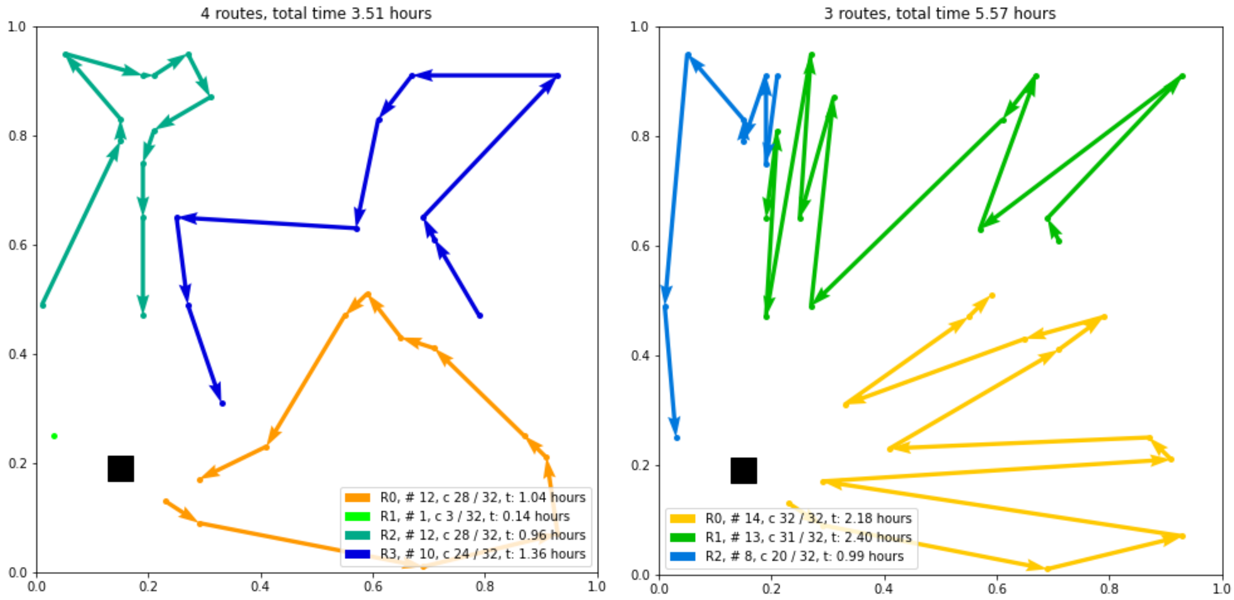

(a) DNN-based Solution (b) Non-DNN Solution

Supplementary Fig. S9: Social distancing policy: passenger capacity=32.

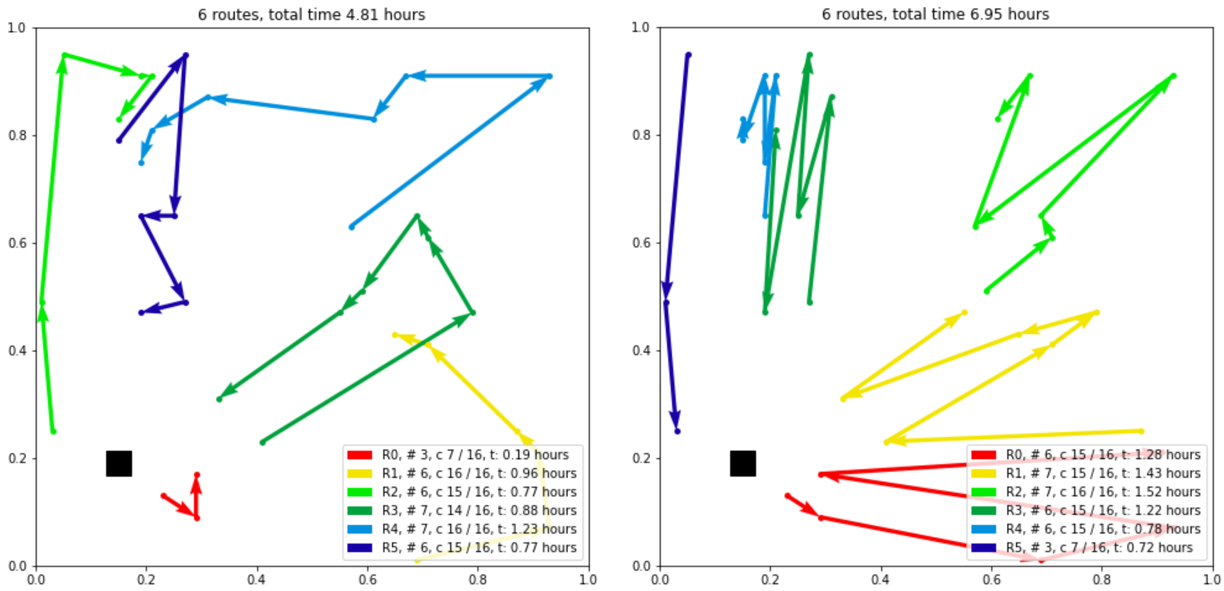

(a) DNN-based Solution (b) Non-DNN Solution

Supplementary Fig. S10: Social distancing policy: passenger capacity=16.

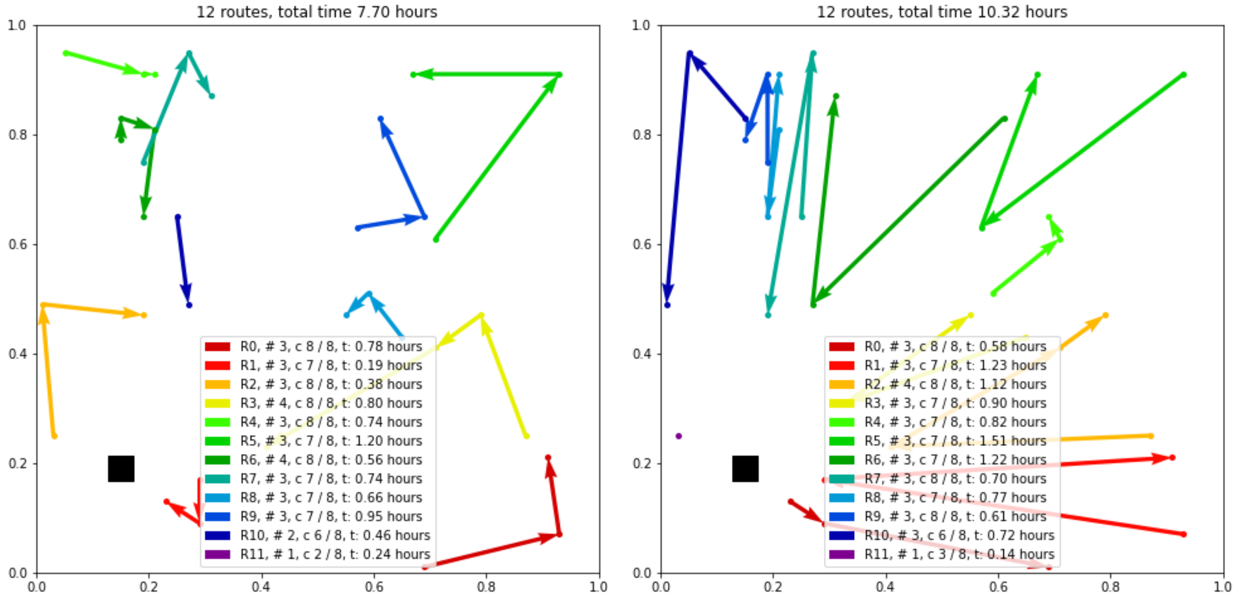

(a) DNN-based Solution (b) Non-DNN Solution

Supplementary Fig. S11: Social distancing policy: passenger capacity=8.

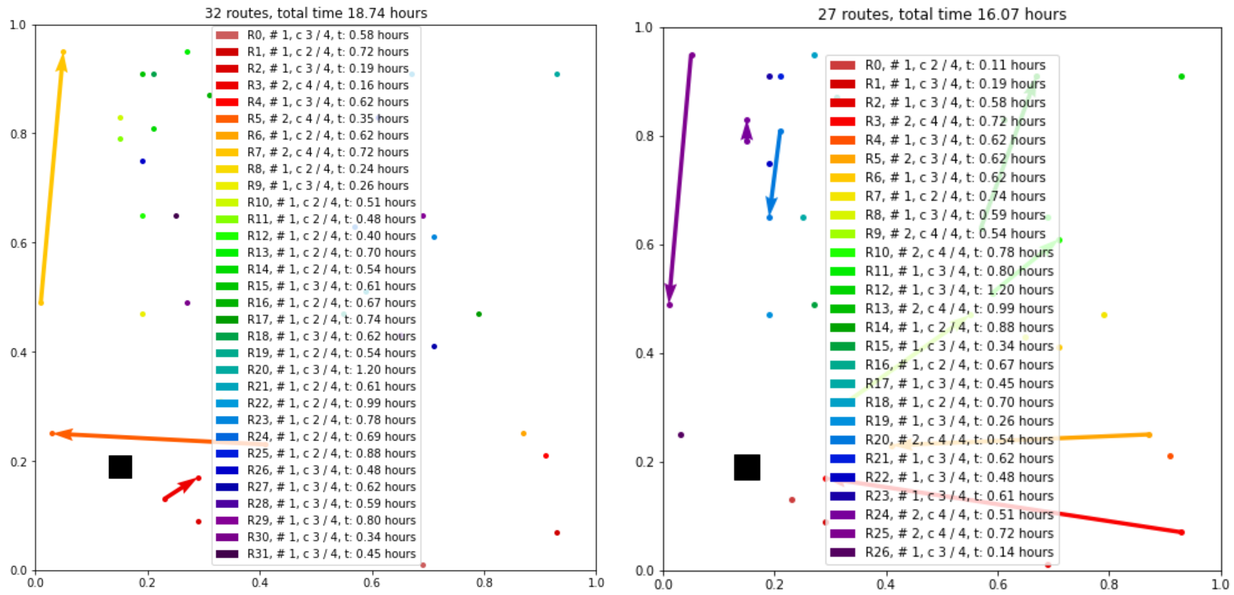

(a) DNN-based Solution (b) Non-DNN Solution

Supplementary Fig. S12: Social distancing policy: passenger capacity=4.

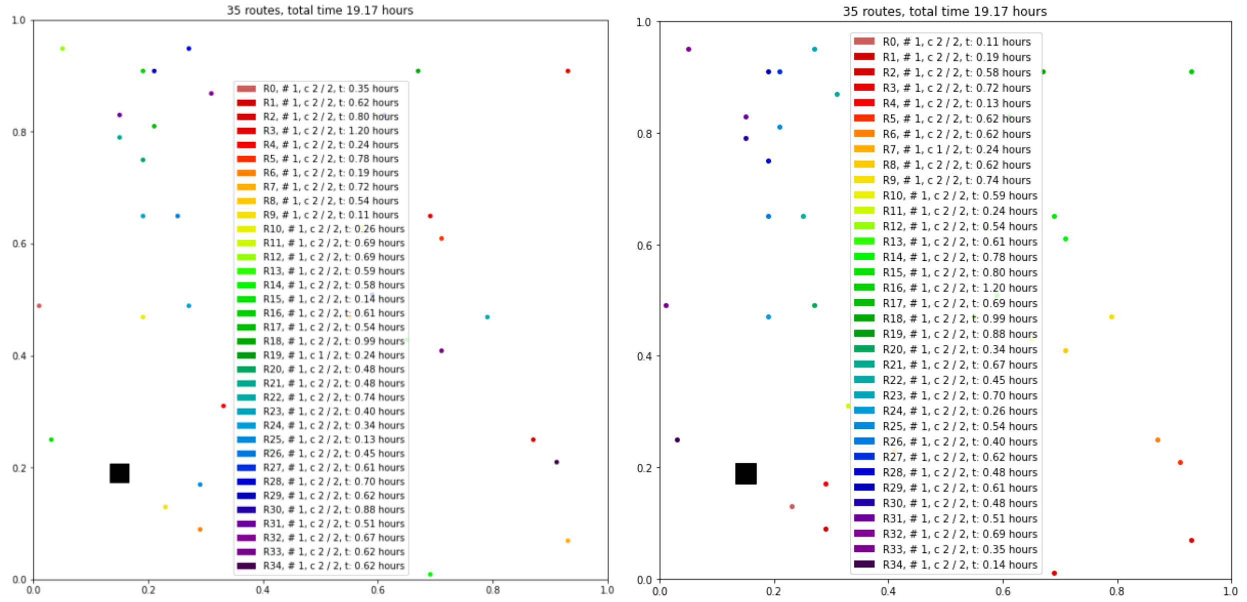

(a) DNN-based Solution (b) Non-DNN Solution

Supplementary Fig. S13: Social distancing policy: passenger capacity=2, Part I.

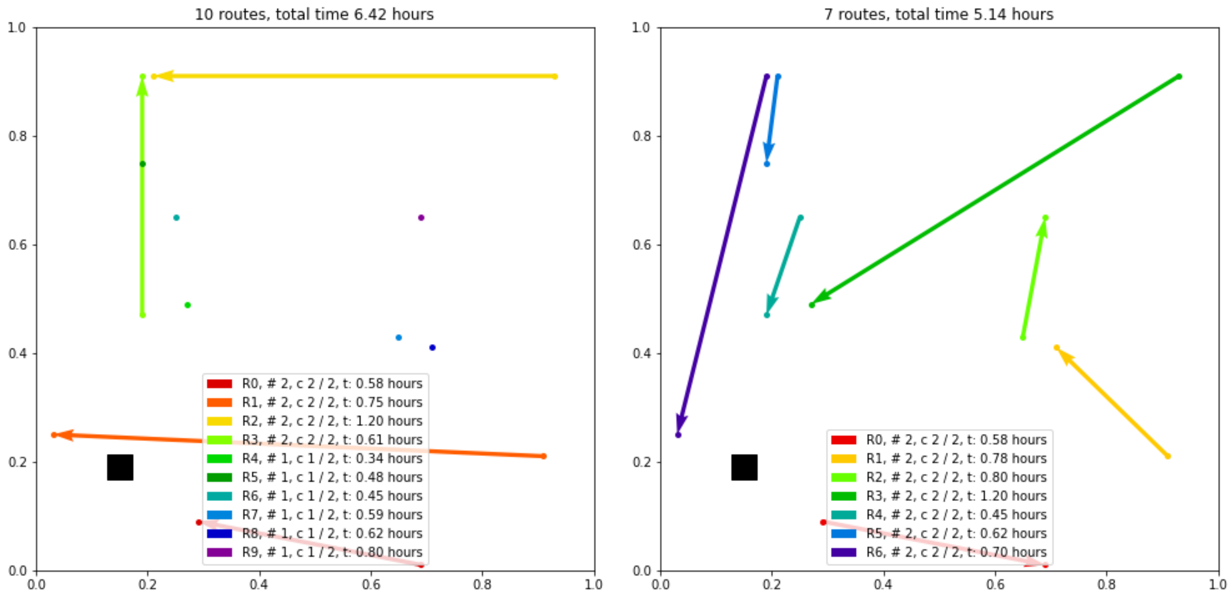

(a) DNN-based Solution (b) Non-DNN Solution

Supplementary Fig. S14: Social distancing policy: passenger capacity=2, Part II.

Dataset 3: 126 people and 52 houses

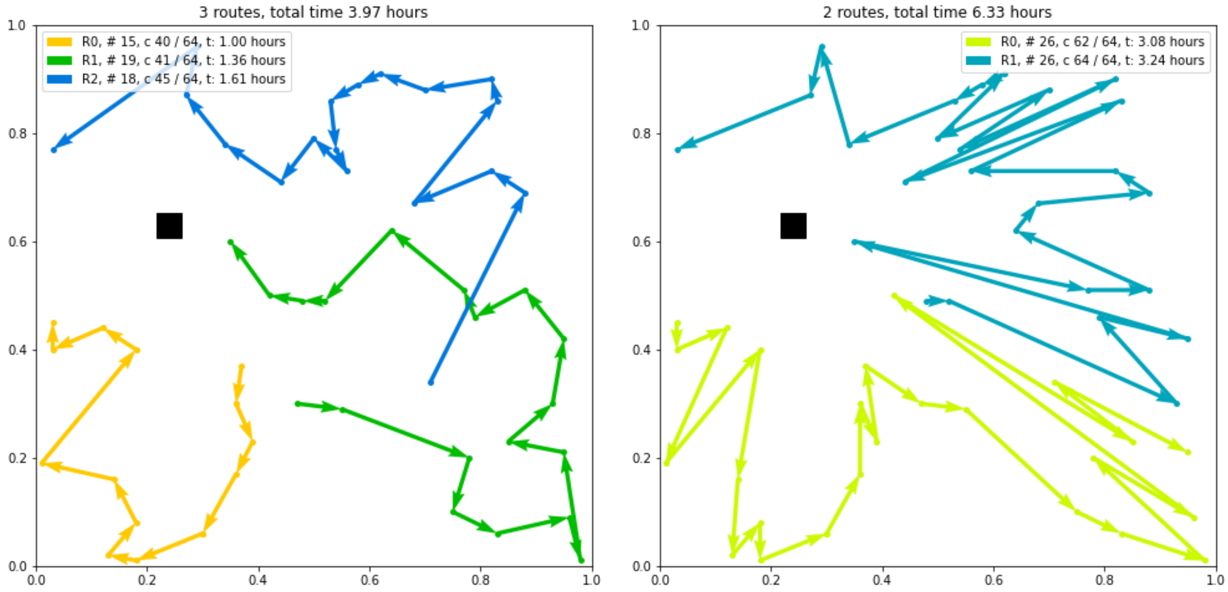

(a) DNN-based Solution (b) Non-DNN Solution

Supplementary Fig. S15: Social distancing policy: passenger capacity=64.

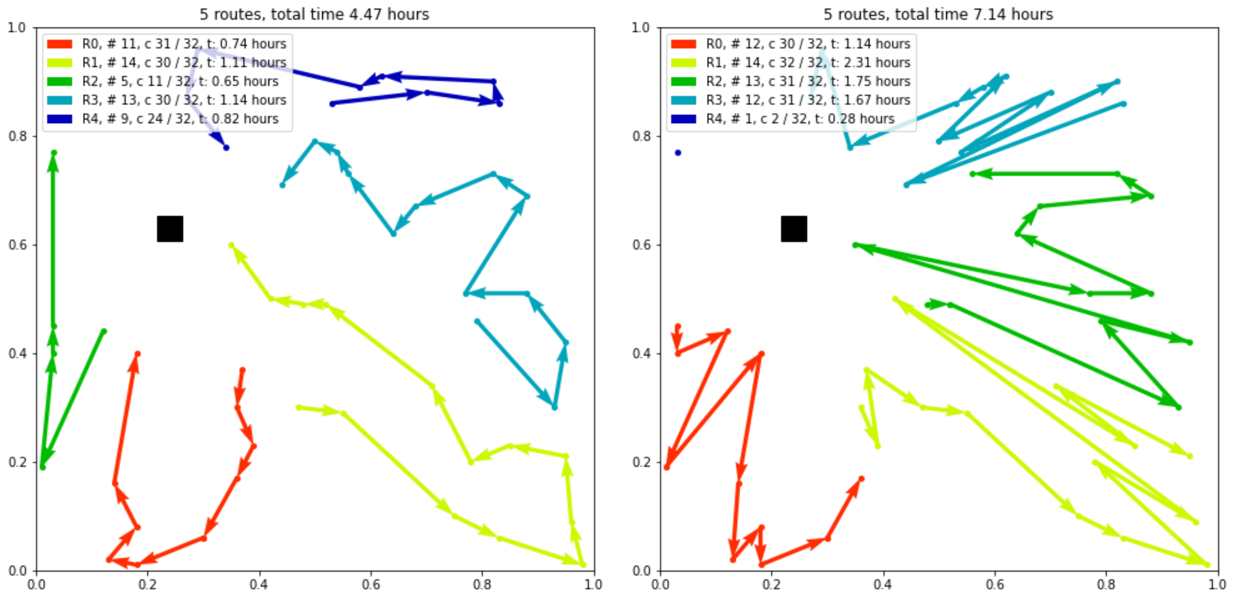

(a) DNN-based Solution (b) Non-DNN Solution

Supplementary Fig. S16: Social distancing policy: passenger capacity=32.

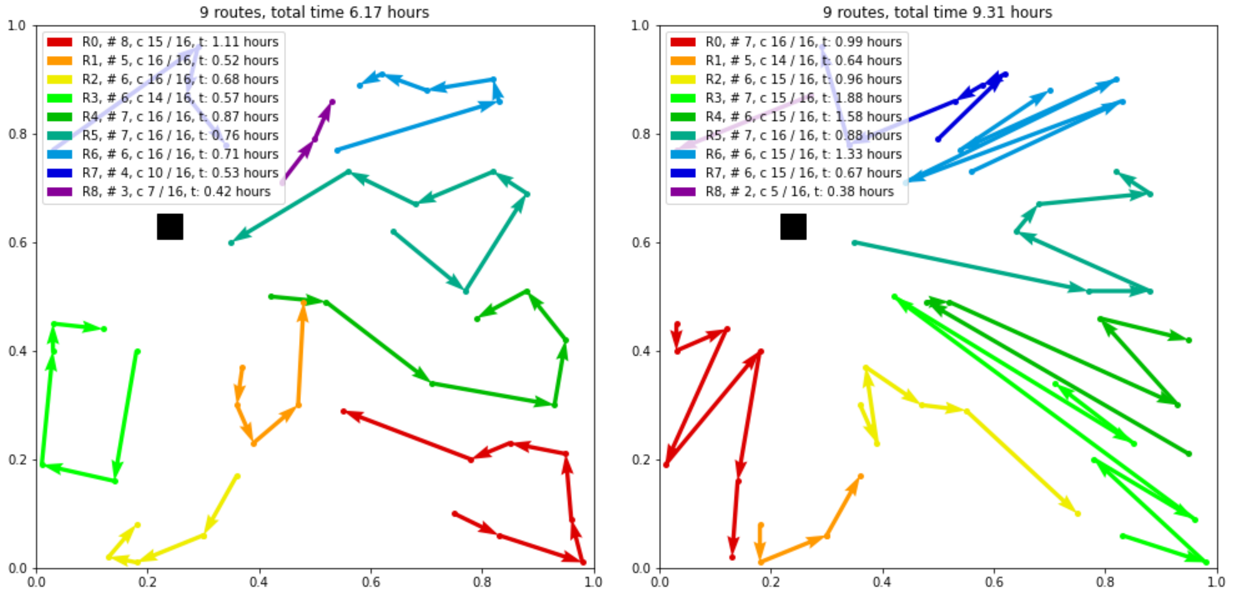

(a) DNN-based Solution (b) Non-DNN Solution

Supplementary Fig. S17: Social distancing policy: passenger capacity=16.

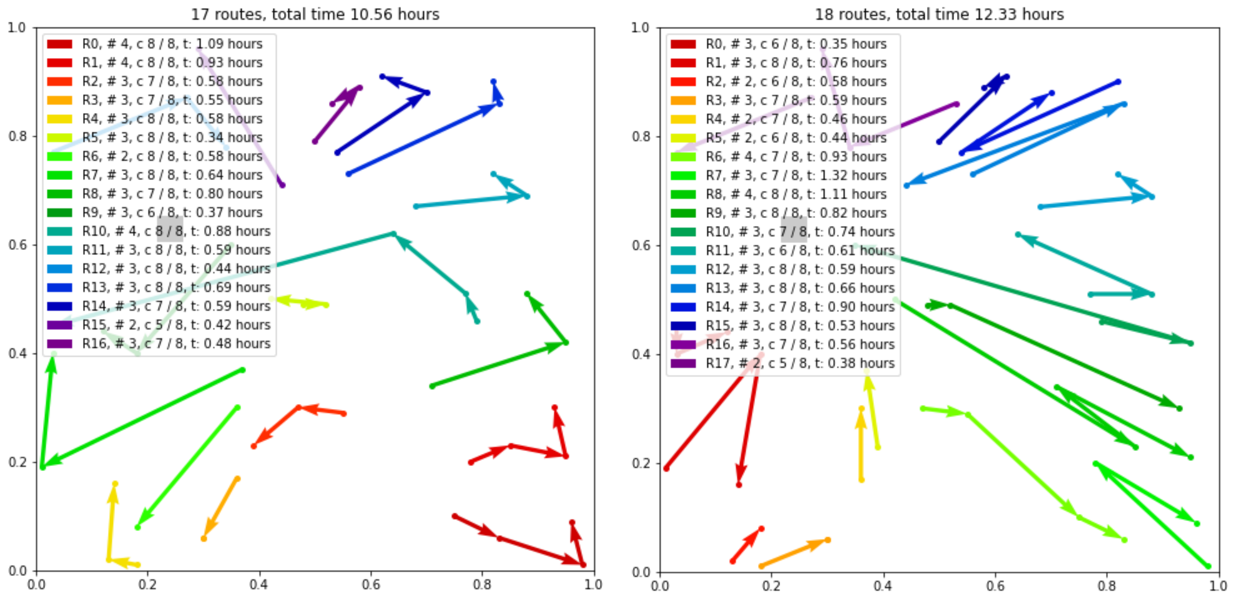

(a) DNN-based Solution (b) Non-DNN Solution

Supplementary Fig. S18: Social distancing policy: passenger capacity=8.

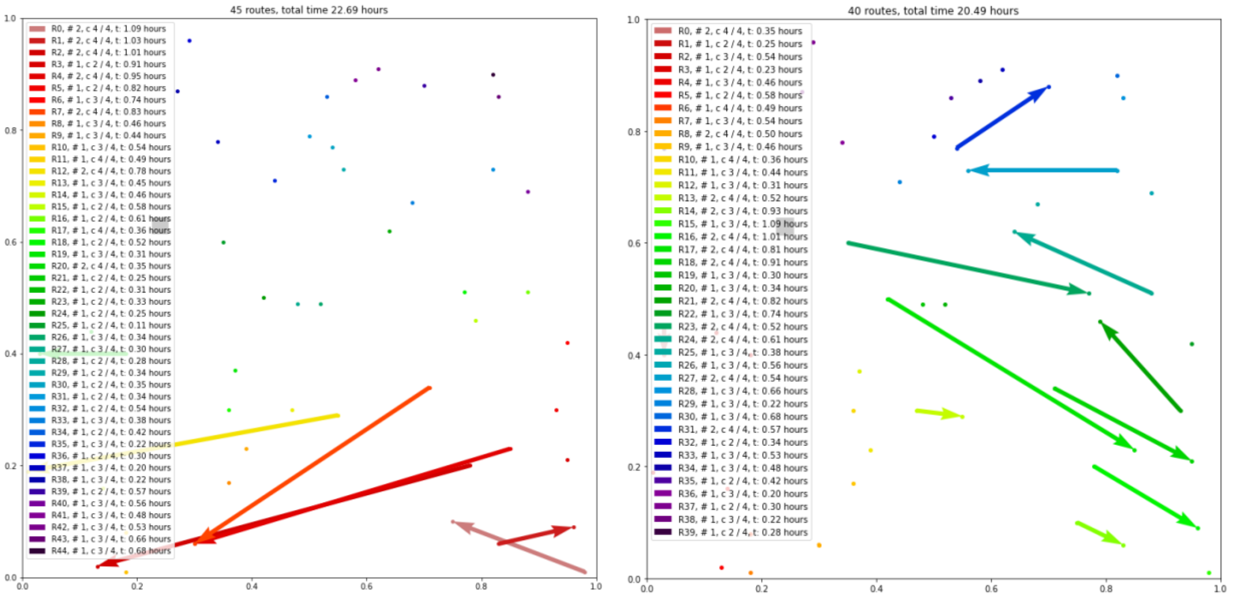

(a) DNN-based Solution (b) Non-DNN Solution

Supplementary Fig. S19: Social distancing policy: passenger capacity=4.

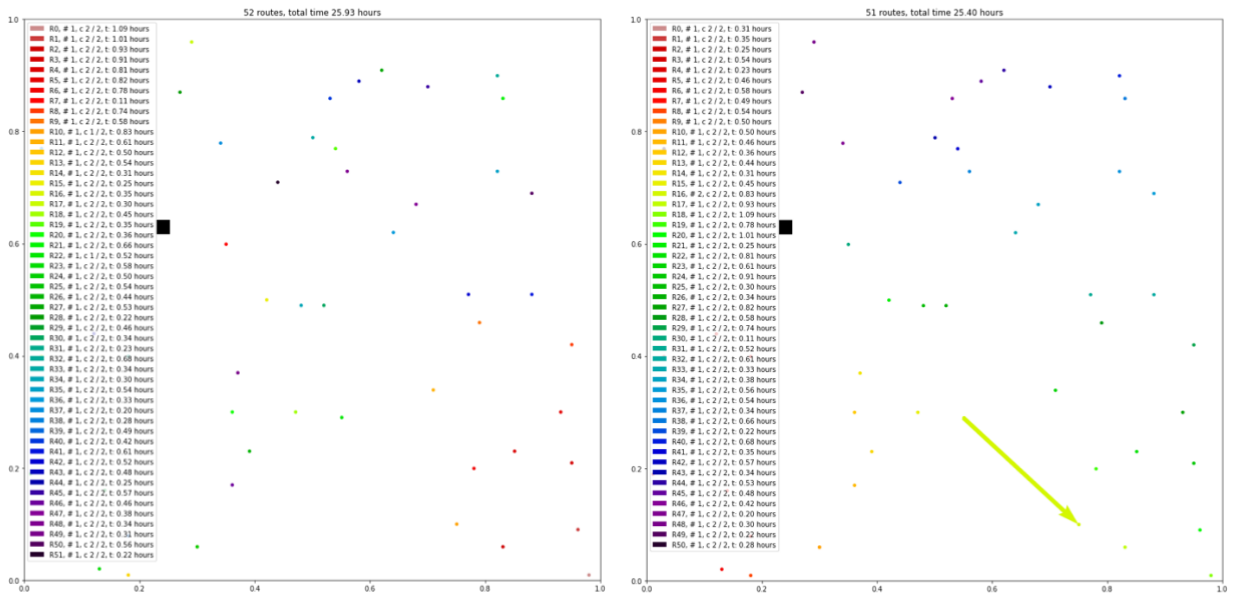

(a) DNN-based Solution (b) Non-DNN Solution

Supplementary Fig. S20: Social distancing policy: passenger capacity=2, Part I.

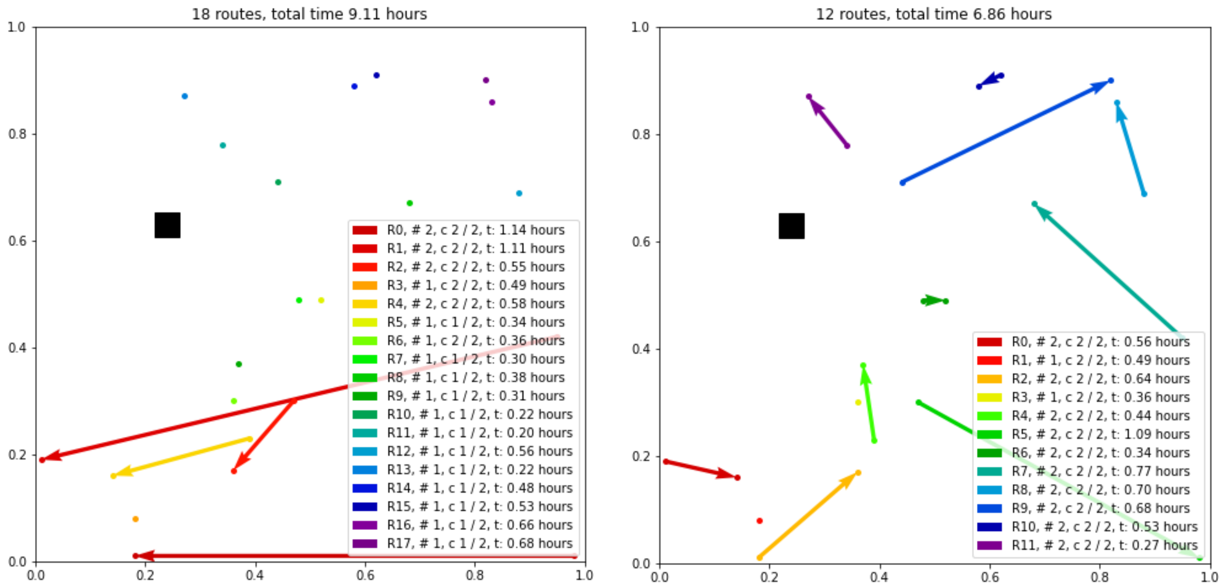

(a) DNN-based Solution (b) Non-DNN Solution

Supplementary Fig. S21: Social distancing policy: passenger capacity=2, Part II.

Dataset 4: 168 people and 68 houses

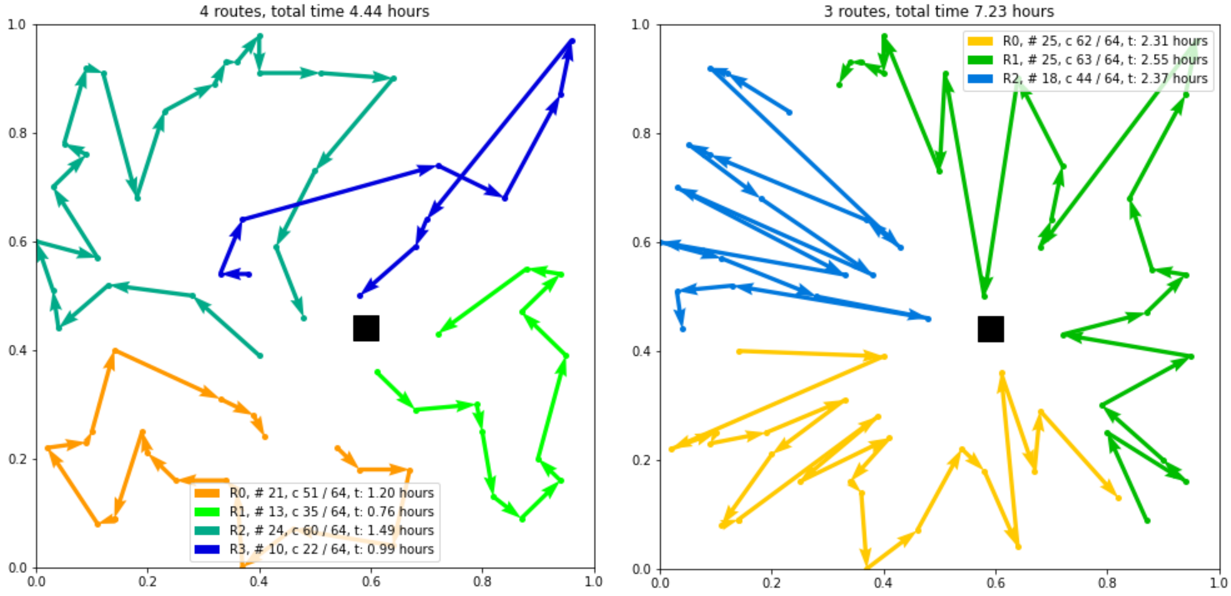

(a) DNN-based Solution (b) Non-DNN Solution

Supplementary Fig. S22: Social distancing policy: passenger capacity=64.

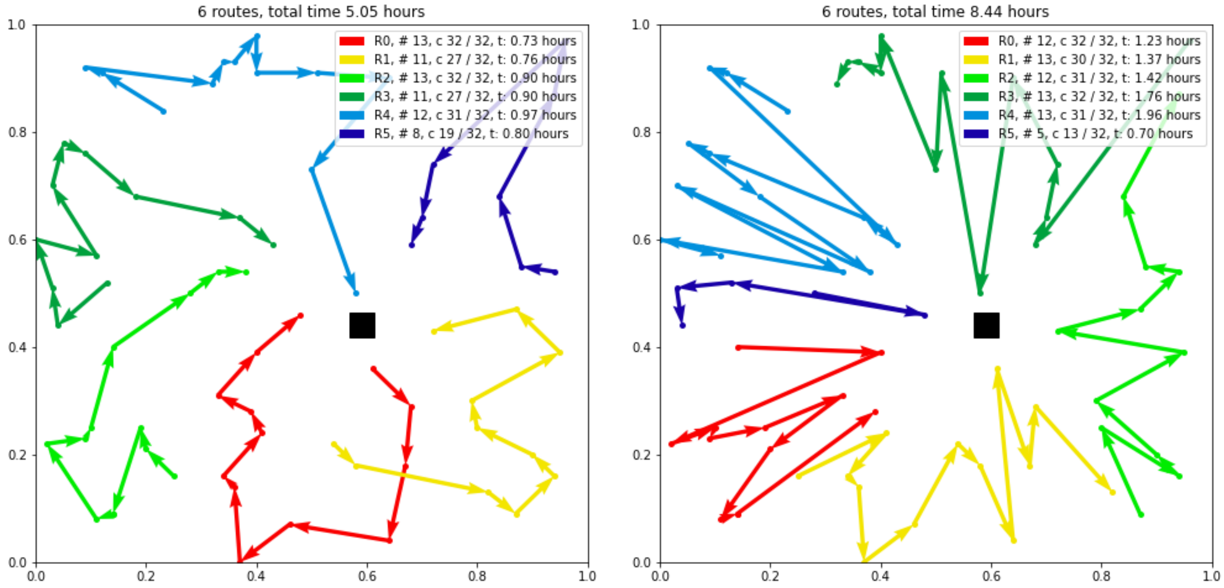

(a) DNN-based Solution (b) Non-DNN Solution

Supplementary Fig. S23: Social distancing policy: passenger capacity=32.

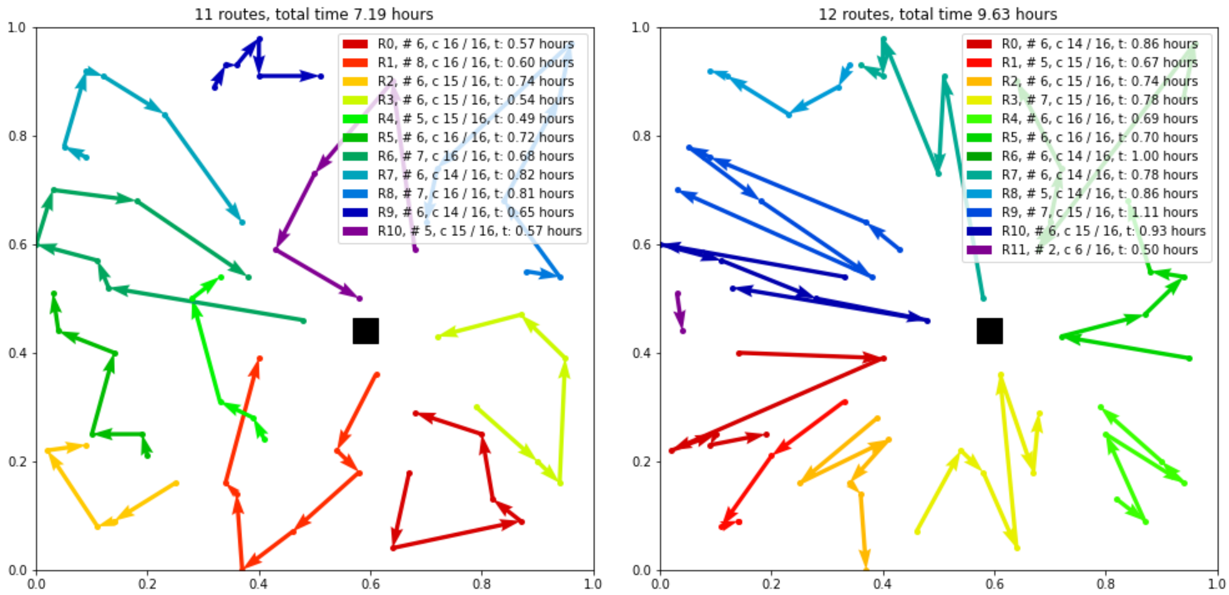

(a) DNN-based Solution (b) Non-DNN Solution

Supplementary Fig. S24: Social distancing policy: passenger capacity=16.

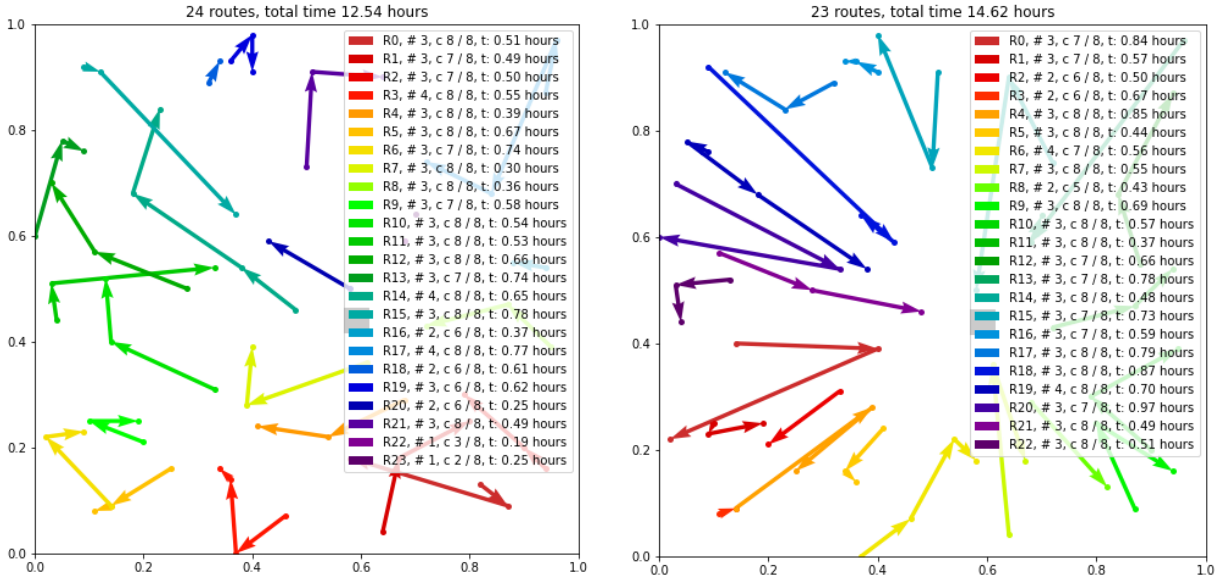

(a) DNN-based Solution (b) Non-DNN Solution

Supplementary Fig. S25: Social distancing policy: passenger capacity=8.

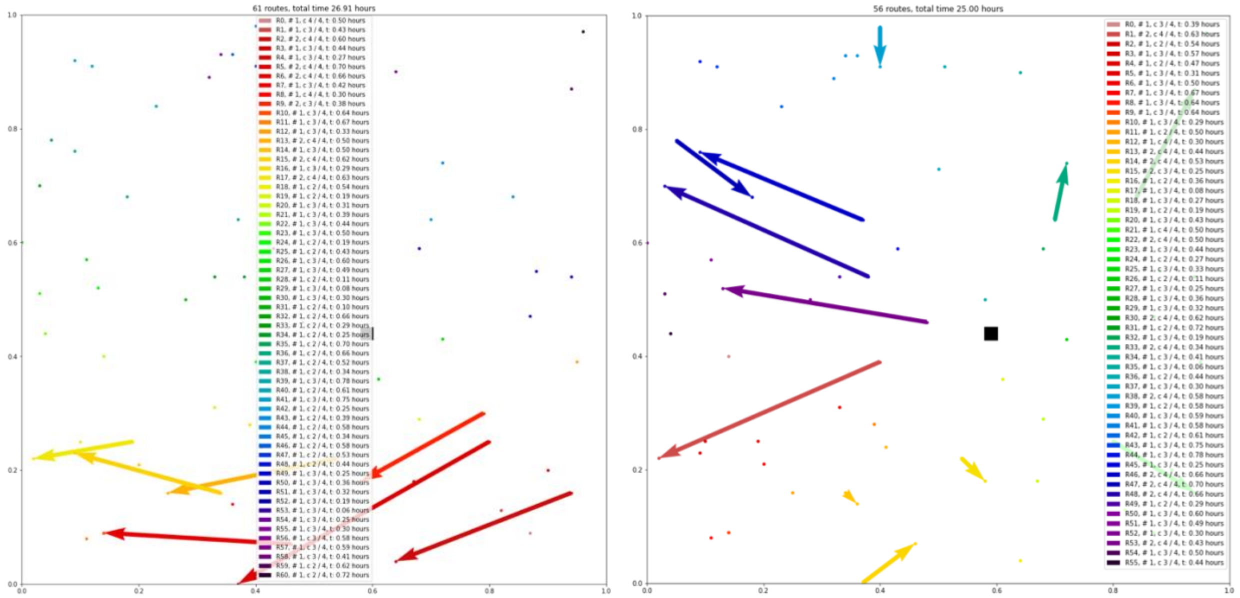

(a) DNN-based Solution (b) Non-DNN Solution

Supplementary Fig. S26: Social distancing policy: passenger capacity=4.

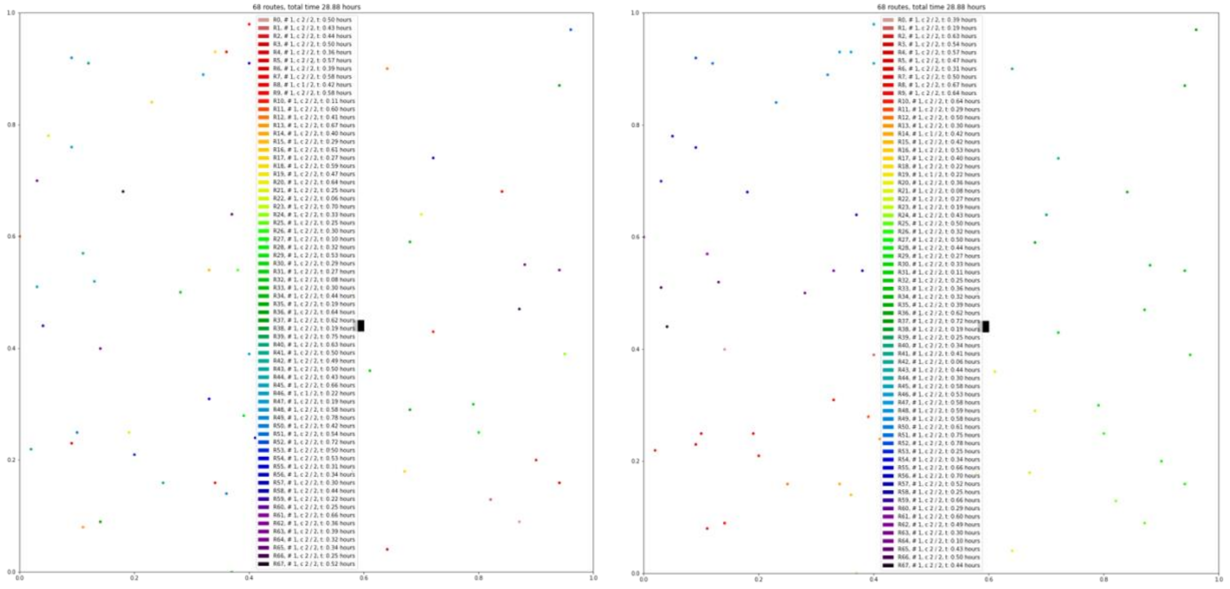

(a) DNN-based Solution (b) Non-DNN Solution

Supplementary Fig. S27: Social distancing policy: passenger capacity=2, Part I.

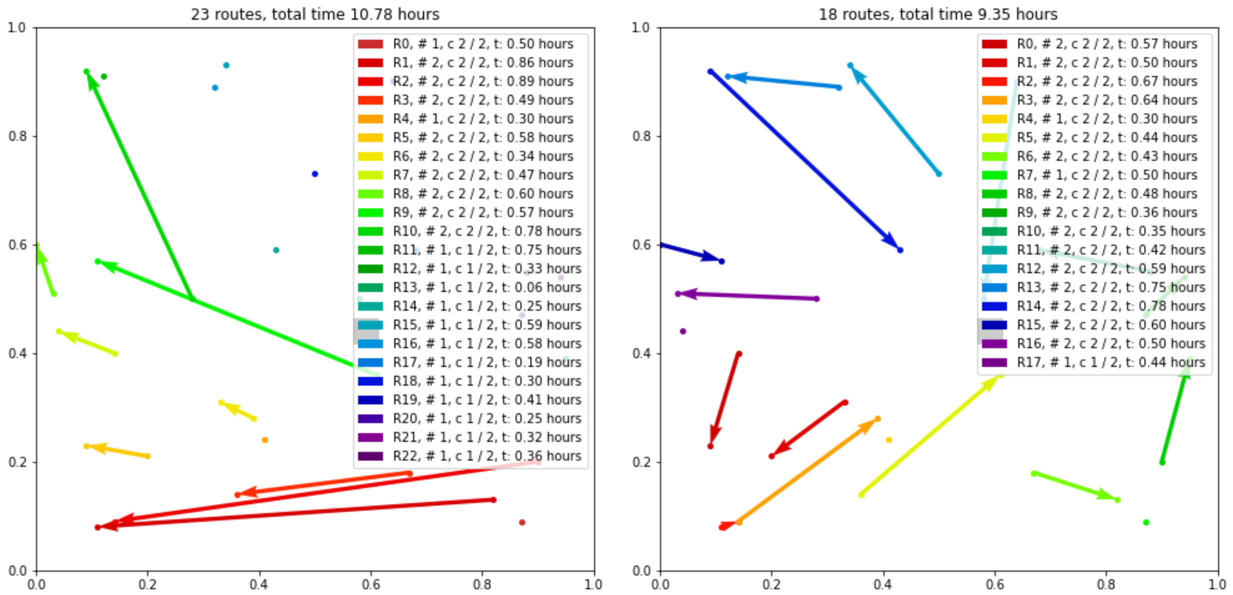

(a) DNN-based Solution (b) Non-DNN Solution

Supplementary Fig. S28: Social distancing policy: passenger capacity=2, Part II.

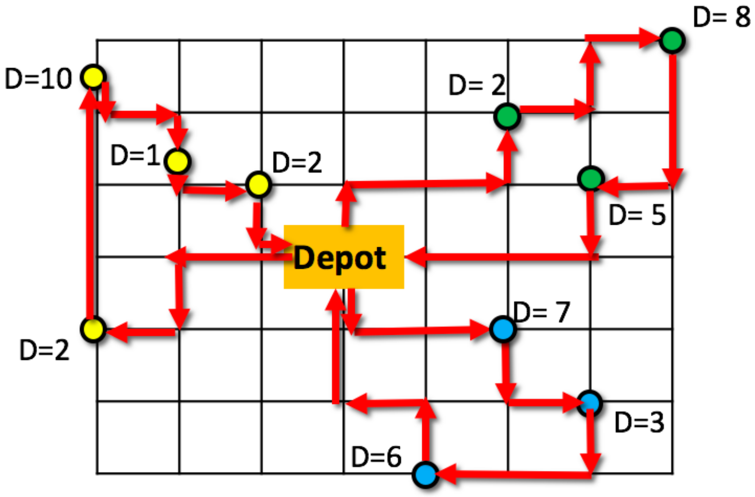

Supplementary Fig. S29: Example plot of a solution to CVRP in Manhattan distance. The vehicle capacity is 16 people per route.
